# Effects of Antiepileptic Drugs, Carbamazepine and Phenytoin, on Plasma Concentrations of Clozapine and Its *N*‐Desmethyl and *N*‐Oxide Metabolites in Japanese Patients With Schizophrenia

**DOI:** 10.1002/npr2.70035

**Published:** 2025-06-26

**Authors:** Kazuro Ikawa, Tomoya Imura, Saori Nakamura, Kanae Murata, Norifumi Morikawa, Mutsumi Sakata, Hidenori Yasuzawa, Naoki Horikawa

**Affiliations:** ^1^ Department of Clinical Pharmacotherapy Hiroshima University Hiroshima Japan; ^2^ Nozoe Hills Hospital Kurume Japan

**Keywords:** carbamazepine, clozapine, drug interaction, phenytoin

## Abstract

Clozapine (CLZ) is used for treatment‐resistant schizophrenia. However, in Japanese patients, its pharmacokinetics are still not well described, and there are no case reports about drug interactions between CLZ and concomitant antiepileptics that can affect cytochrome P450 (CYP) activity, because CLZ is mainly metabolized by CYP. In two Japanese cases, for therapeutic drug monitoring, the trough plasma concentrations of CLZ, *N*‐desmethyl clozapine (NCLZ) and *N*‐oxide clozapine (OCLZ) were measured, and the ratios of them and the daily dose (D) were used as indicators of drug metabolism. In a male patient in his 20s, the CLZ/D ratio ([ng/mL]/[mg/day]) was significantly decreased, and both NCLZ/CLZ and OCLZ/CLZ ratios were significantly increased with carbamazepine, relative to CLZ alone. In another male patient in his 30s, the CLZ/D ratio was lower with phenytoin than without phenytoin. The NCLZ/CLZ ratio was higher with phenytoin than without phenytoin. Despite these expectations, the OCLZ/CLZ ratio showed a slight decrease with phenytoin. These results in the two Japanese cases demonstrate the effects of antiepileptics on plasma concentrations, not only of CLZ but also of its major metabolites NCLZ and OCLZ. Metabolism‐enhancing interactions with CLZ were demonstrated for carbamazepine, a CYP3A4‐ and CYP1A2‐inducer. However, for phenytoin, which is also known as a CYP3A4‐inducer, the drug interactions with CLZ were not very clear due to the small limited data. These pharmacokinetic findings in only two cases must be confirmed by further investigations.

## Abstract 2

In Case 1, metabolism‐enhancing effects of carbamazepine on clozapine pharmacokinetics were demonstrated. However, in Case 2, the effects of phenytoin were not very clear due to the small limited data.

## Graphical abstract

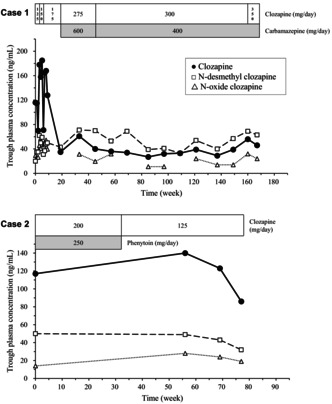

## 1 Introduction

Clozapine (CLZ) is the only antipsychotic drug for treatment‐resistant schizophrenia [1]. In Japan, CLZ tablets (Clozaril 25 mg and 100 mg) have been clinically used since July 2009 [2]. The concentration of CLZ has been shown to correlate with its efficacy and safety. Therapeutic drug monitoring has been implemented in Japan for insurance coverage since April 2022. However, the pharmacokinetics of CLZ in Japanese patients have not been well described. Because CLZ is mainly metabolized by hepatic cytochrome P450 (CYP), CYP‐mediated drug interactions can occur. In particular, antiepileptic drugs that can affect CYP activity are often used concomitantly for schizophrenia patients in clinical practice, and drug interactions with CLZ have been reported [3, 4, 5, 6, 7, 8]. However, in Japanese patients, there are no case reports about interactions between CLZ and antiepileptics. Additionally, the interactions have usually been reported only for unchanged CLZ and not for its major metabolites [3, 4, 5, 6, 8]. In this report, we present the cases of two Japanese patients to examine the effects of antiepileptic carbamazepine and phenytoin, known as CYP inducers, on plasma concentrations of CLZ and its major metabolites *N*‐desmethyl clozapine (NCLZ) and *N*‐oxide clozapine (OCLZ).

## 2 Materials and Methods

### 2.1 Measurement of CLZ, NCLZ, and OCLZ Concentrations in Plasma

Venous blood was collected before the next dose of CLZ (usually 10–14 h after the last dose). Blood samples were centrifuged and trough concentrations was measured in the resulting plasma.

The plasma concentrations of CLZ, NCLZ, and OCLZ were simultaneously measured using reversed‐phase high‐performance liquid chromatography, according to the methods of Avenoso et al. [9] with minor modifications. Briefly, plasma samples (600 μL) were deproteinized by adding NaOH (0.5 M) and hexane:3‐methyl‐1‐butanol (75:25 [v/v]), using triprolidine as an internal standard. The mixture was vortexed and centrifuged, and the resulting upper layer was added for extraction to phosphate buffer (0.1 M, pH 2.2) and diethyl ether. After vortexing and centrifugation, the bottom layer (20 μL) was injected into a chromatograph, separated on a C6 column (40°C) and detected by ultraviolet absorbance (254 nm). The mobile phase was phosphate buffer (0.06 M, pH 2.7) containing sodium 1‐heptanesulfonate:acetonitrile (70:30 [v/v]) at a flow rate of 1 mL/min. The lower limits of quantification were 10 ng/mL, and the calibration curves were linear up to 1500 ng/mL for CLZ, NCLZ, and OCLZ. In intraday and interday assays, accuracy (as absolute error from 100%) and precision (as coefficient of variation) values were within 10% for all analytes.

### 2.2 Analysis of Pharmacokinetic Data

EZR, an R‐based statistical analysis package [10], was used for data analysis. The Mann–Whitney U test was used for comparisons between the CLZ monotherapy and concomitant therapy with antiepileptics groups.

## 3 Case Presentation

### 3.1 Case 1

A Japanese man in his 20s (height 170 cm, average weight 64.9 kg, nonsmoker) was diagnosed with treatment‐resistant schizophrenia. Clozapine was initiated, and carbamazepine was concomitantly started to control agitation, aggression, and dysphoria. Although eszopiclone (2 mg) was used before bedtime for the treatment of insomnia, this coadministration may not affect CYP activities [11].

**FIGURE 1:**
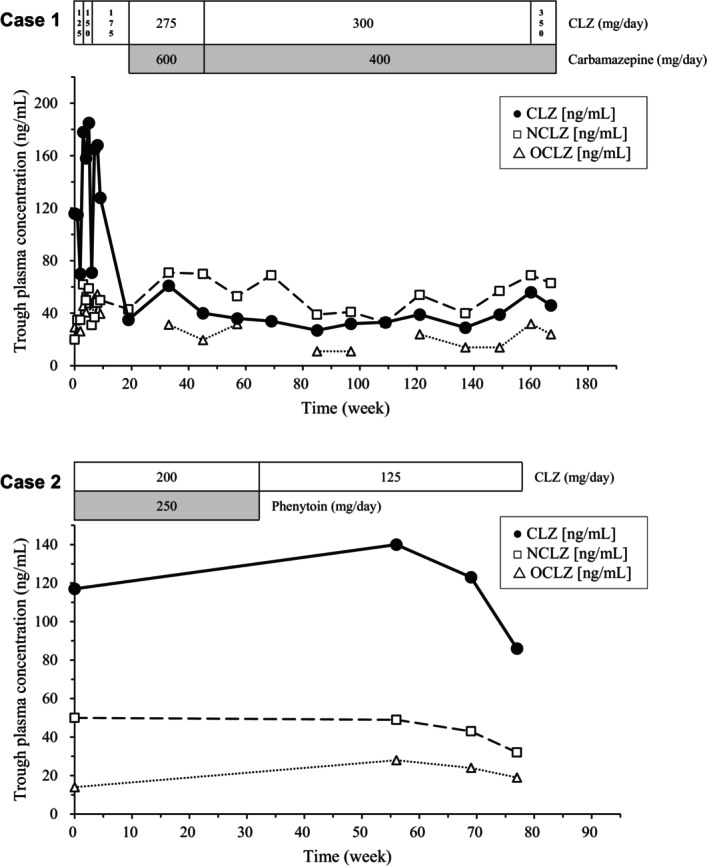
Trough plasma concentrations [ng/mL] of clozapine (CLZ), *N*‐ desmethyl clozapine (NCLZ) and *N*‐oxide clozapine (OCLZ) after the start of therapeutic drug monitoring in Case 1 with carbamazepine and Case 2 with phenytoin.

Figure 1 shows the changes in plasma concentrations of CLZ, NCLZ, and OCLZ after the start of therapeutic drug monitoring. The CLZ concentrations decreased from 128 ng/mL (CLZ 175 mg/day alone) to 35 ng/mL (CLZ 275 mg/day after the initiation of carbamazepine 600 mg/day). The NCLZ concentrations increased from 43 ng/mL to 71 ng/mL with carbamazepine.

Table 1 shows the ratios of the daily dose (D), CLZ, NCLZ, and OCLZ, as indicators of drug metabolism. The CLZ/D ratio ([ng/mL]/[mg/day]) was, on average, 6.97 times lower with carbamazepine (0.129 ± 0.033) than without carbamazepine (0.899 ± 0.247). The NCLZ/CLZ ratio was, on average, 4.24 times higher with carbamazepine (1.400 ± 0.261) than without carbamazepine (0.330 ± 0.096). Likewise, OCLZ/CLZ ratio was, on average, 1.58 times higher with carbamazepine (0.494 ± 0.159) than without carbamazepine (0.313 ± 0.069).

**TABLE 1:** Ratios of the daily dose (D) (mg/day) and trough plasma concentrations (ng/mL) of clozapine (CLZ), *N‐* desmethyl clozapine (NCLZ), and *N*‐oxide clozapine (OCLZ).

	CLZ/D ([ng/mL]/[mg/day])	NCLZ/CLZ	OCLZ/CLZ
Case 1			
CLZ alone (10 points)	0.899 ± 0.247	0.330 ± 0.096	0.313 ± 0.069
CLZ + carbamazepine (13 points)	0.129 ± 0.033*	1.400 ± 0.261*	0.494 ± 0.159*
Case 2			
CLZ alone (3 points)	0.931 ± 0.221	0.357 ± 0.013	0.194 ± 0.006
CLZ + phenytoin (1 point)	0.585	0.427	0.120

*Note:* Mean ± standard deviation.

* Significant difference (*p* < 0.01) relative to CLZ alone.

### 3.2 Case 2

A Japanese man in his 30s (height 186 cm, average weight 98.3 kg, nonsmoker) was diagnosed with treatment‐resistant schizophrenia. Clozapine was concomitantly administered with phenytoin to control agitation, aggression, and dysphoria. Although flurazepam (30 mg) was used before bedtime for the treatment of insomnia, this co‐administration may not affect CYP activities [12].

As shown in Figure 1, CLZ concentrations increased from 117 ng/mL (CLZ 200 mg/day with phenytoin 250 mg/day) to 140 ng/mL (CLZ 125 mg/day alone in week 56). However, NCLZ concentration was almost the same as the concentration with phenytoin (50 ng/mL) and without phenytoin (49 ng/mL in week 56). The drug concentrations were lower in week 77; this reason was unclear, but one possibility may be due to the later sampling time (18.7 h) than others (14.2 h in week 56 and 14.9 h in week 69).

As shown in Table 1, CLZ/D ratio was, on average, 1.59 times lower with phenytoin (0.585) than without phenytoin (0.931 ± 0.221). The NCLZ/CLZ ratio was, on average, 1.20 times higher with phenytoin (0.427) than without phenytoin (0.357 ± 0.013). In contrast, OCLZ/CLZ ratio showed a slight decrease with phenytoin (0.120) from those without phenytoin (0.194 ± 0.006).

## 4 Discussion

We demonstrate the pharmacokinetic interactions between CLZ and antiepileptic drugs. These interactions resulted in plasma concentration changes not only in unchanged CLZ but also in its metabolites. To the best of our knowledge, this is also the first case report on interactions between CLZ and antiepileptics in Japanese patients with schizophrenia.

In Case 1, with carbamazepine, CLZ/D ratio was decreased significantly (*p* < 0.01), and both NCLZ/CLZ and OCLZ/CLZ ratios were increased significantly (*p* < 0.01), relative to CLZ alone. These results suggest that concomitant use of carbamazepine decreases CLZ concentrations due to enhanced metabolism, which is consistent with earlier reports. In one report, 2 cases [3] showed increased CLZ concentrations after discontinuation of carbamazepine. In two other reports, 2 cases [4] and 6 cases [5] showed increased CLZ concentrations after switching from carbamazepine to oxcarbazepine. The CLZ/D ratio was lower in the carbamazepine‐concomitant group than in the CLZ monotherapy group [6]. Regarding the metabolites, there is a report of 1 case [7] in which both NCLZ concentration and NCLZ/CLZ ratio increased after switching from valproic acid to carbamazepine. However, OCLZ was not measured or analyzed in any cases with carbamazepine.

Plasma concentration after administration of CLZ was reported to be highest with unchanged CLZ, followed by the metabolites NCLZ and OCLZ (average ratio of CLZ:NCLZ:OCLZ = 1:0.59:0.19) [13]. In the metabolism from CLZ to NCLZ, its major contributors CYP3A4 and CYP1A2 were reported to account for approximately 22% and 30% of the metabolism, respectively [14]. From CLZ to OCLZ, its major contributors CYP3A4 and CYP1A2 were reported to account for approximately 75% and 15%, respectively [15]. Carbamazepine is generally known to be primarily a CYP3A4 inducer and secondarily a CYP1A2 inducer [16, 17]. Both CYP3A4‐ and CYP1A2‐inducing effects are considered to be responsible for large increases not only in NCLZ/CLZ ratio but also in OCLZ/CLZ ratio.

In Case 2, with phenytoin, the CLZ/D ratio showed a decrease and the NCLZ/CLZ ratio showed an increase (both without statistical significance), relative to CLZ alone. These results suggest that the concomitant use of phenytoin can decrease CLZ concentrations due to enhanced metabolism, which is consistent with an earlier report [8]. In the report, 2 cases showed a 2.81‐ to 5.51‐fold decrease in the CLZ/D ratio after administration of phenytoin (300 mg/day). Regarding the metabolites, there are no case reports describing the administration of phenytoin in which NCLZ and OCLZ were measured or analyzed. Theoretically, phenytoin can increase the OCLZ/CLZ ratio. Contrary to the expectations in Case 2, the OCLZ/CLZ ratio showed a slight decrease with phenytoin, relative to CLZ alone. This may be due to the small number of samples (especially just only one time point with phenytoin) and the long interval between the periods with and without phenytoin, which cannot exclude any possibility of changes in other factors.

Phenytoin is generally known as a CYP3A4 inducer [17] but there is no strong evidence to prove that it is a CYP1A2 inducer, implying that phenytoin has fewer effects than carbamazepine. In Case 2, effects of phenytoin on CLZ/D ratio were observed, but the decreases were not large (1.59‐fold) and could not be clearly explained by the pharmacokinetic behavior of NCLZ and OCLZ. Additionally, with the exception of the two reported cases [8], no studies have investigated interactions between CLZ and phenytoin. Interactions with CLZ are mentioned in the Japanese package insert of carbamazepine [18] but not in that of phenytoin [19]. Therefore, we consider that phenytoin causes some interactions with CLZ, but it may be less important than those caused by carbamazepine. Further studies are thus needed to clarify the degree of drug interactions with phenytoin and its clinical significance.

## 5 Conclusion

The effects of antiepileptic drugs on plasma concentrations, not only of CLZ but also of its major metabolites NCLZ and OCLZ, were presented in two Japanese schizophrenia cases. For carbamazepine, metabolism‐enhancing interactions with CLZ were demonstrated, emphasizing the need for careful monitoring and dose adjustment of CLZ when coadministered with carbamazepine. However, for phenytoin, the drug interactions with CLZ was not very clear due to the small limited data. Further investigations are required to verify and generalize these pharmacokinetic findings obtained from only two cases.

## Author Contributions

K.I. analyzed the pharmacokinetic data and wrote the first draft of manuscript. T.I., S.N., and K.M. measured plasma concentrations of clozapine. N.M., and N.H. checked the pharmacokinetic analysis and refined the manuscript. M.S., H.Y., and N.H. were involved in treating the cases and collecting and interpreting the data. All authors read, commented on, and improved the manuscript and approved the final version for submission.

## Ethics Statement

The study was approved by the Ethics Committee of Nozoe Hills Hospital, Kurume, Japan and performed in accordance with the Japanese Ethical Guidelines for Medical and Health Research Involving Human Subjects.

## Consent

All patients provided written informed consent.

## Conflicts of Interest

The authors declare no conflicts of interest.

## Data Availability

Essential data are already shown in this report. In terms of ethical concerns, no more detailed data are provided to protect personal information.
